# Haplotype Phasing and Inheritance of Copy Number Variants in Nuclear Families

**DOI:** 10.1371/journal.pone.0122713

**Published:** 2015-04-08

**Authors:** Priit Palta, Lauris Kaplinski, Liina Nagirnaja, Andres Veidenberg, Märt Möls, Mari Nelis, Tõnu Esko, Andres Metspalu, Maris Laan, Maido Remm

**Affiliations:** 1 Department of Bioinformatics, Institute of Molecular and Cell Biology, University of Tartu, Tartu, Estonia; 2 Institute for Molecular Medicine Finland (FIMM), University of Helsinki, Helsinki, Finland; 3 Estonian Biocentre, Tartu, Estonia; 4 Human Molecular Genetics Research Group, Institute of Molecular and Cell Biology, University of Tartu, Tartu, Estonia; 5 Institute of Biotechnology, University of Helsinki, Helsinki, Finland; 6 Chair of Mathematical Statistics, Institute of Mathematical Statistics, University of Tartu, Tartu, Estonia; 7 The Estonian Genome Center of University of Tartu, Tartu, Estonia; 8 Department of Biotechnology, Institute of Molecular and Cell Biology, University of Tartu, Tartu, Estonia; 9 Division of Endocrinology, Boston Children’s Hospital, Boston, Massachusetts, United States of America; 10 Department of Genetics, Harvard Medical School, Boston, Massachusetts, United States of America; 11 Broad Institute, Cambridge, Massachusetts, United States of America; Seoul National University College of Medicine, REPUBLIC OF KOREA

## Abstract

DNA copy number variants (CNVs) that alter the copy number of a particular DNA segment in the genome play an important role in human phenotypic variability and disease susceptibility. A number of CNVs overlapping with genes have been shown to confer risk to a variety of human diseases thus highlighting the relevance of addressing the variability of CNVs at a higher resolution. So far, it has not been possible to deterministically infer the allelic composition of different haplotypes present within the CNV regions. We have developed a novel computational method, called *PiCNV*, which enables to resolve the haplotype sequence composition within CNV regions in nuclear families based on SNP genotyping microarray data. The algorithm allows to i) phase normal and CNV-carrying haplotypes in the copy number variable regions, ii) resolve the allelic copies of rearranged DNA sequence within the haplotypes and iii) infer the heritability of identified haplotypes in trios or larger nuclear families. To our knowledge this is the first program available that can deterministically phase null, mono-, di-, tri- and tetraploid genotypes in CNV loci. We applied our method to study the composition and inheritance of haplotypes in CNV regions of 30 HapMap Yoruban trios and 34 Estonian families. For 93.6% of the CNV loci, *PiCNV* enabled to unambiguously phase normal and CNV-carrying haplotypes and follow their transmission in the corresponding families. Furthermore, allelic composition analysis identified the co-occurrence of alternative allelic copies within 66.7% of haplotypes carrying copy number gains. We also observed less frequent transmission of CNV-carrying haplotypes from parents to children compared to normal haplotypes and identified an emergence of several *de novo* deletions and duplications in the offspring.

## Introduction

DNA copy number variation is a subtype of structural genetic variation that may increase the copy number of a particular DNA segment from normal two copies per diploid genome (diploid copy number, CN = 2) to more than two (diploid CN>2; duplication, triplication, etc.) or decrease to less than two (CN<2; deletion) copies. In the human genome, CNV lengths typically range from few kilo-bases to several mega-bases. It is estimated that they cumulatively cover at least 5% of the human genome [1–4] and suggested that they play a major role in different human phenotypic traits, including disease susceptibility [5, 6]. CNVs can overlap with genes and, by changing DNA methylation and gene expression patterns or altering coding sequences of these genes, have functional and phenotypic consequences [7–9]. In their high resolution study of HapMap individuals, Conrad *et al*. found that as much as 13% of RefSeq genes overlapped validated CNVs [3].

A multitude of phenotypes and diseases have already been robustly associated with CNVs (reviewed in References [10–12]) and many disease-associated copy number variants of high penetrance are known [13–16]. However, the penetrance is not complete for most CNV loci associated so far [17–20], possibly due to epigenetic modifications, other genetic variants in the vicinity, modifier genes and regulatory elements (reviewed by Cooper *et al*.) [21], and potentially also due to allele dosage effects in combination with alternative allelic copies present within the CNV regions. It is plausible that variable allele dosage of other types of genetic variants with directly functional effect (e.g. SNPs—single nucleotide variants in coding sequence) found within the copy number altered DNA segments could modulate the severity of a certain phenotype. So far, majority of studies have disregarded the allelic nature of CNVs and have only considered the total estimated number of copies on two homologous chromosomes (CN = 0, 1, 2, 3, etc.) [19, 22–25] or the continuous normalised microarray intensity data [8, 26, 27].

**Fig 1:**
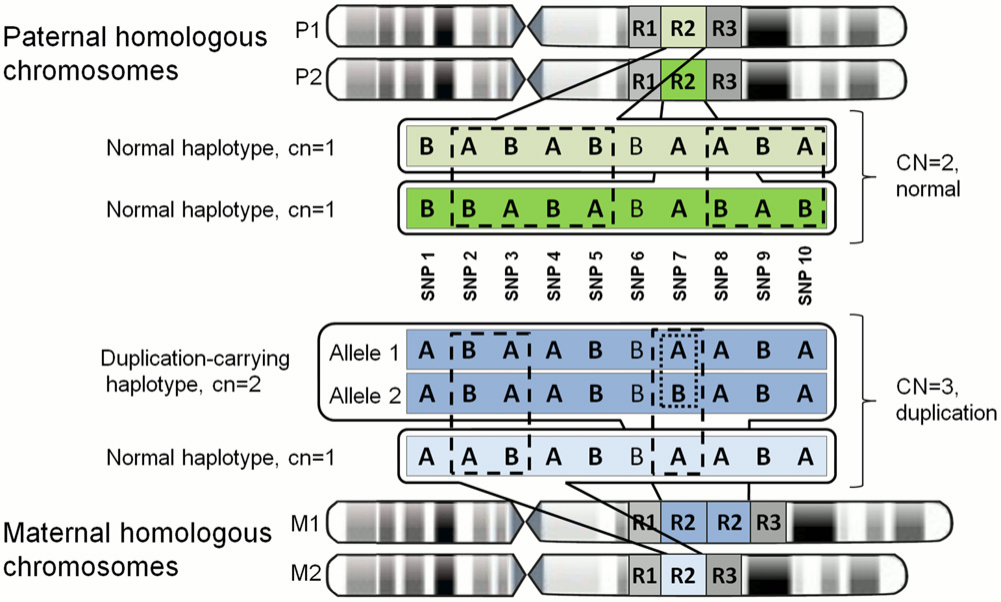
Phasing and allelic composition of normal and CNV-carrying haplotypes on parental homologous chromosomes. A chromosomal region involving copy number variation is denoted with ‘R2’. In the given example, father is the carrier of two normal haplotypes of ‘R2’ on chromosomes P1 and P2 (diploid copy number of ‘R2’, CN = 2), whereas mother has a combination of a duplication-carrying (on M1) and normal (M2) haplotypes (diploid copy number of ‘R2’, CN = 3). Haplotype-informative SNP genotypes in ‘R2’ sequence that can be used for phasing and determining the parental origin (in offspring) of given normal and CNV-carrying haplotypes are given in bold letters and genotypes that are polymorphic *between* normal or duplication-carrying parental haplotypes are indicated with dashed rectangles. The duplication-carrying haplotype on maternal M1 chromosome is composed of two allelic copies of the sequence ‘R2’ distinguished by genotype variability at position SNP7 (polymorphic SNP variant *within* the duplication-carrying haplotype), indicated with dotted rectangle.

The ability to phase and differentiate between normal haplotypes (with haploid copy number of 1; cn = 1) and CNV-carrying haplotypes (haploid copy number cn = 0 or cn>1) with different allelic composition (Fig 1) along with their parental origin would enable new, possibly more specific and therefore more powerful association analyses with phenotypic traits [6, 28]. By ‘normal haplotype’ we hereinafter denote a continuous chromosomal segment carrying one copy of a certain unique sequence per homologous chromosome and by ‘CNV-carrying haplotype’ we denote a continuous chromosomal region carrying no or more than one copy of a certain (otherwise unique) sequence per homologous chromosome. Just as the phase information has been shown to be important in case of SNP variants [29, 30], elucidating the exact distribution of copies of a particular DNA sequence on two sets of chromosomes may prove beneficial in case of CNVs [6, 31].

In addition to the accustomed two-letter SNP genotypes, the genome-wide genotyping microarray data allows to infer null, mono-, tri- and tetraploid ‘CNV genotypes’ [32–35] and perform phasing of the exact haplotypes within each CNV region. In spite of the fact that there are several methods and algorithms that can statistically phase CNV-carrying haplotypes and infer chromosome-specific copy number [36–43], there are no computational methods available that would also enable deterministic phasing of the exact allelic composition of haplotypes in CNV regions of studied individuals. In order to further improve CNV-based studies, it would be of utmost importance to develop new methods and algorithms that could accurately infer the chromosome-specific copy number and also allelic composition at CNV regions [5, 31, 44–46].

In the current study, we developed a computational method *PiCNV* for phasing of normal and variant-carrying haplotypes within CNV regions to be applied on genome-wide data of trios and larger nuclear families. We aimed to determine, how often it is unambiguously possible to infer the exact haplotypes and alternative allelic copies within CNV regions and follow their transmission in nuclear families. Exact haplotype phasing within CNV regions also allowed us to study the inheritance patterns between normal and CNV-carrying haplotypes and between haplotypes carrying deletions and duplications.

## Results

### *PiCNV*, computational method for phasing normal and CNV-carrying haplotypes of CNV regions in families

**Fig 2:**
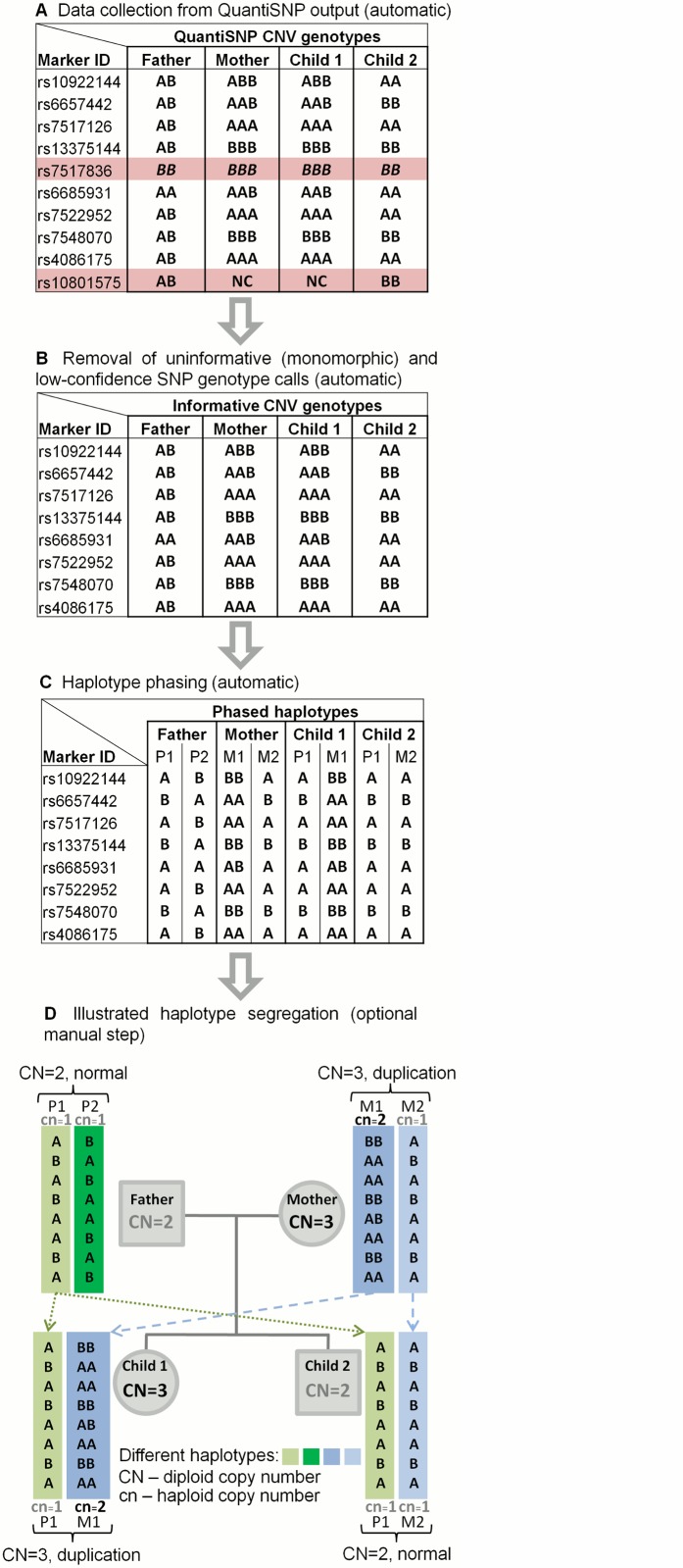
Computational phasing of normal and CNV-carrying haplotypes. (**A**) First, CNV and regular two-letter genotypes are collected from the QuantiSNP output for each family member at a locus of interest. (**B**) Next, markers that have any low-confidence genotype calls or the call could not have been made (‘NC’ genotypes, e.g. marker rs10801575, marked with the red background) and monomorphic markers that are not informative for haplotype phasing in the studied region (e.g. marker rs7517836; marked with the red background) are filtered out. (**C**) Informative high-confidence genotypes are then phased considering all family members simultaneously and the resulting haplotypes are presented as the result. (**D**) The family tree of these phased haplotypes can be further visualised for the corresponding CNV region.

We have developed a computational method that can deterministically phase normal (cn = 1) and CNV-carrying (deletion, cn = 0; or copy number gain, cn>1) haplotypes in CNV regions in nuclear families based on genotype and copy number estimates from SNP microarray data (Fig 1). Our algorithm (called *PiCNV* for ‘Phasing and inheritance of Copy Number Variants’) works with the parsed output of widely used CNV calling algorithms like PennCNV [47], QuantiSNP [33] and Fawkes [34]. In addition to CNV calling from genotyping microarray data, these algorithms can infer the diploid allelic composition (referred to as ‘CNV genotype’ or ‘CNV-based SNP genotype’) for each SNP marker within CNV regions. As regular two-letter genotypes (e.g. ‘AA’ or ‘AB’), these null, mono-, tri- and tetraploid genotypes (e.g. ‘–‘, ‘A’, ‘ABB’ and ‘ABBB’, respectively) are inferred from B-allele frequency (BAF) data and represent the total allelic composition from both homologous chromosomes of a studied individual [32–34, 48]. By using CNV calls, haplotype-informative genotypes (polymorphic in the parents) and user-defined family structure, our algorithm will phase the allelic composition within each CNV region in studied families (Figs 1 and 2).

**Fig 3:**
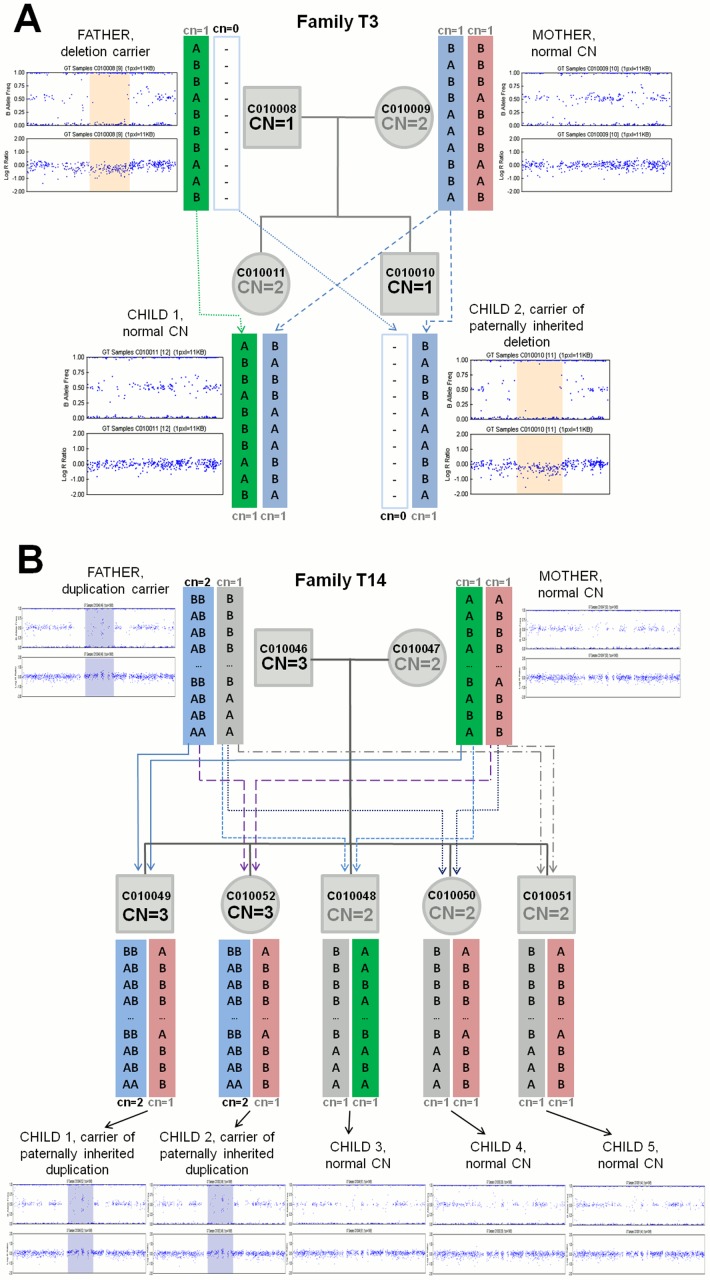
Examples of unambiguously phased CNV regions involving deletion- and duplication-carrying haplotypes in families. (**A**) Inherited 820 kb-long deletion on chromosome 16:15369798–16190572 in family T3. A deletion-carrying haplotype (cn = 0) is inherited from father (C010008) to son (Child 2, C010010). The daughter (Child 1, C010011) has inherited normal haplotypes (cn = 1) from both parents. (**B**) Inherited 166 kb-long duplication on chromosome 10:47007374–47173619 in family T14. A duplication-carrying haplotype (cn = 2) is inherited from father (C010046) to one son (Child 1, C010049) and daughter (Child 2, C010052). All other children have inherited normal haplotypes (cn = 1) from both parents. Coloured arrows show the transmission of specific haplotypes from parents to offspring in a given CNV region. Respective B-allele frequency (BAF, upper panel) and total fluorescent signal intensity (Log R Ratio—LRR, lower panel) plots from Illumina Genome Viewer are shown next to the parents and each child.

*PiCNV* works by examining consecutive regular (two-letter) and CNV genotypes in each family member in a CNV region present in any member of the corresponding family. It will test all possible haplotypes and their transmission according to all Mendelian inheritance scenarios in the studied CNV locus in a given family. If the family includes more than one child, all children will be considered simultaneously in this step and conclusively, for each CNV locus, *PiCNV* will select these normal and deletion- or duplication-carrying haplotypes and transmission scenarios that can explain the allelic composition for every member of the corresponding family (Fig 3). In case it is not possible to explain the allelic composition in an offspring by Mendelian transmission of parental haplotypes, non-Mendelian transmission scenarios—*de novo* deletion/duplication events and uniparental isodisomy/heterodisomy are automatically considered. Provided informative genotypes are present in parental haplotypes, *PiCNV* is also able to determine on which parental chromosome the *de novo* event has occurred. Complete source code and Linux binaries for *PiCNV* are available from http://bioinfo.ut.ee/picnv.

### Testing of *PiCNV*—CNV calling and confirmation in two microarray datasets

To test our phasing methodology and *PiCNV* algorithm, we analysed two family-based datasets (Table 1). First, we analysed publicly available high-resolution microarray (Illumina Human1M) data generated by Itsara *et al*. [49] for 30 HapMap Yoruban (HapMap YRI) mother-father-child trios from the International HapMap Project [50, 51]. Microarray data were analysed with QuantiSNP [33] and PennCNV [47] algorithms to call CNVs (S1 Fig). These calls were merged (as intersection of overlapping QuantiSNP and PennCNV calls) for each individual and only CNVs that were similarly called (same type overlapping variant calls) were considered. In order to achieve high-quality CNV dataset, these CNV calls were further confirmed with an independent set of validated CNV calls from custom Affymetrix high-resolution microarrays for the same Yoruban individuals [52]. Only CNVs that were similarly called in the same individuals in both datasets were considered and further converged into 1364 CNV regions in families that were used in the following analyses (S2 Table). In each such CNV region, at least one member of the corresponding family had a confirmed CNV while family members with no CNVs were proven to have no variant calls in any of the datasets.

**Table 1:** Detailed description of analysed family-based SNP microarray datasets.

Dataset	HapMap YRI	EGCUT
**Description**	Yoruba Nigerian family trios from the International HapMap Project	Estonian families from population-based cohort from the Estonian Genome Center
**Total number of families**	30	34
**Number of family trios**	30	22
**Number of larger families**	-	8 quartets, 2 quintets, 1 sextet and 1 septet
**Number of individuals**	90	121
**Source of genomic DNA**	Lymphoblastoid cell lines	Peripheral blood
**SNP genotyping platform**	Illumina Human1M (from Itsara *et al*., Reference [49])	Illumina HumanCNV370K
**Number of CNVs called**	4307	1088
**CNV confirmation**	Affymetrix custom high-resolution microarray data (from Matsuzaki *et al*., Reference [52])	Visual confirmation in Illumina Genome Viewer; qRT-PCR for 20 visually confirmed CNV regions
**Confirmed CNVs (CNVRs)**	2067 (1364)	246 (146)

Secondly, we analysed low-resolution microarray (Illumina HumanCNV370K) data for 34 Estonian families from the Estonian Genome Center of University of Tartu (EGCUT dataset) consisting of 22 mother-father-child trios and 12 larger families (Table 1). CNVs were called as in the HapMap YRI data, converged in families and further visually confirmed from the microarray signal intensity data. This resulted in 146 visually confirmed CNV regions in families that were used in the following analyses (S2 Table). Out of 146 CNV regions, 12 randomly selected and 8 putative *de novo* CNV regions were validated by quantitative real-time PCR in all members of the respective families (S1 Table). According to these validation experiments, the false positive rate of CNV calling was estimated at 11% and false negative rate at 9% for the EGCUT CNV dataset.

### Computational phasing of CNV regions in HapMap YRI and EGCUT datasets

Based on the occurrences of CNVs among parents in each CNV region and family, we divided the CNV regions and the corresponding transmission events into three groups (S1 Fig). Firstly, all CNV regions with only one parent carrying a CNV (irrespective whether it was transmitted to their offspring or not) were assigned to group A. Secondly, all CNV regions where both parents were carriers of a CNV in the same locus were assigned to group B. Finally, all putative *de novo* CNVs with no carriers among the parents but with at least one child as a carrier of a CNV in the corresponding locus were assigned to group C.

**Fig 4:**
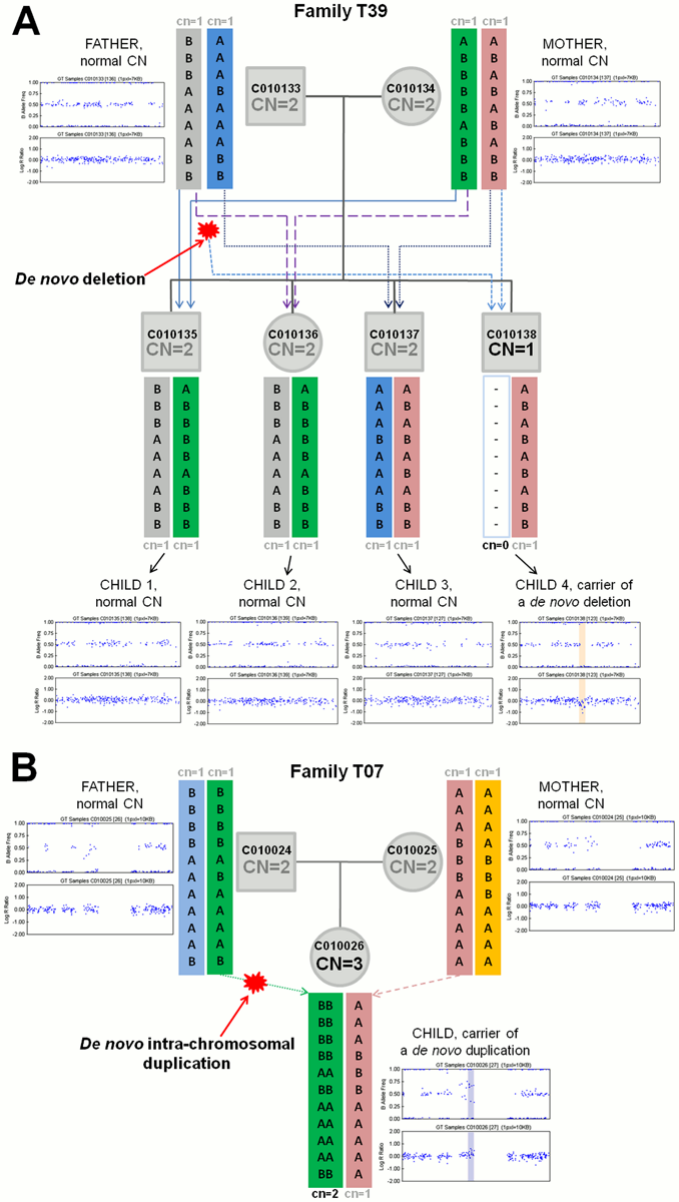
Examples of *de novo* copy-number variants in offspring. (**A**) *De novo* arisen 67 kb-long deletion on chromosome 6:80596173–80663256 in family T39. Children 1–3 (C010135, C010136 and C010137) have inherited one normal haplotype from both parents. One child (Child 4, C010138) has inherited one normal haplotype from his mother (C010134) and a paternal haplotype with a *de novo* deletion event in the corresponding region. (**B**) *De novo* arisen 167 kb-long duplication on chromosome 2:110175122–110331912 in family T07. The only child (C010026) has inherited one normal haplotype from her mother (C010025) and a paternal haplotype with a *de novo* intra-chromosomal duplication event in the corresponding region. Coloured arrows show the transmission of specific haplotypes from parents to offspring in a given CNV region. Respective B-allele frequency (BAF, upper panel) and total fluorescent signal intensity (Log R Ratio—LRR, lower panel) plots from Illumina Genome Viewer are shown next to the parents and each child.

The subsequent three groups of CNV regions were subjected to haplotype phasing using the *PiCNV* program in both HapMap YRI and EGCUT datasets combined. The *PiCNV* was able to unambiguously phase 93.6% (1414 out of 1510) of all CNV regions and automatically determine the distribution of normal and deletion or duplication-carrying haplotypes in parents and their offspring (Table 2; Figs 3 and 4). The unambiguous phasing efficiency was the highest in group A of CNV regions, reaching 96.3% (1366 out of 1418; Table 2). All remaining CNV regions (3.7%; 52 out of 1418) in group A were duplication CNV regions (CN≥3) containing only uninformative monomorphic genotypes (e.g. ‘AA’ or ‘AAA’, etc.) or CNV probes that do not interrogate any SNP variants. Subsequently, *PiCNV* was not able to unambiguously distinguish between exact maternal and paternal haplotypes and/or follow their transmission in offspring, resulting in several equally possible Mendelian transmissions in the corresponding families (S2a Fig). Similar limitations were also observed in several CNV regions in groups B and C resulting in unambiguous phasing in 60% and 33.3% of CNV regions in those groups, respectively (Table 2). In groups B and C, higher phasing efficiency was observed for deletion CNV loci and in larger families (data not shown). In the remaining CNV regions of groups B and C, it was not possible to unambiguously determine the underlying haplotypes and/or follow their transmission due to the combination of complex CNVs (multi-copy parental CNVs) and/or presence of only uninformative SNP and CNV genotypes, resulting in several equally possible Mendelian or non-Mendelian transmissions (e.g. *de novo* duplication or uniparental heterodisomy) in the corresponding loci and families (S2b and S2d Fig).

**Table 2:** Unambiguously phased CNV regions in families.

Dataset	HapMap YRI	EGCUT	Combined
**All regions**	**94.4%** (1287/1364)	**87%** (127/146)	**93.6%** (1414/1510)
**Group A**	**97.2%** (1251/1287)	**87.8%** (115/131)	**96.3%** (1366/1418)
**Group B**	**55.4%** (31/56)	**88.9%** (8/9)	**60%** (39/65)
**Group C**	**23.8%** (5/21)	**66.7%** (4/6)	**33.3%** (9/27)

Percentage of CNV regions in HapMap YRI and EGCUT families where *PiCNV* was automatically able to unambiguously determine the underlying normal and CNV-carrying haplotypes in parents and follow their transmission in offspring. Group A—CNV regions where only one parent had a CNV; group B—CNV regions where both parents had a CNV in the same locus; group C—putative *de novo* CNV regions.

### Transmission of normal and CNV-carrying haplotypes

In order to determine the transmission rate of normal (cn = 1) and CNV-carrying (cn = 0 or cn>1) haplotypes, we then analysed all 1366 CNV regions of HapMap YRI and EGCUT datasets in group A, where it was unambiguously possible to determine all parental haplotypes and follow their segregation in the offspring (Fig 3). By counting the number of transmission events of normal or CNV-carrying haplotypes from the parents carrying a CNV, we found that CNV-carrying haplotypes had been transmitted slightly less frequently than normal haplotypes. In the HapMap YRI dataset, 580 (46.4%) of such unambiguously phased transmission events corresponded to the inheritance of the CNV-carrying haplotype, whereas 671 (53.6%) of the transmissions involved the normal haplotype (p-value = 0.011). Similar trend was observed in the EGCUT dataset with 70 (41.7%) transmissions of the variant-carrying haplotype and 98 (58.3%) events of inheritance of the normal haplotype (p-value = 0.037).

To further investigate this tendency, we analysed the transmission of normal and deletion/duplication-carrying haplotypes (in HapMap YRI and EGCUT group A datasets combined) while considering CNV length (Table 3). And although this analysis revealed small deviations from the expected Mendelian transmission rate of 50% in nearly all CNV length intervals for both deletions and duplications, the previously observed differences were mainly driven by the under-transmission of short (<10kb) deletion-carrying haplotypes with transmission rate of 44.1%. Although statistically not significant, similar tendency of lower transmission rate was observed for longer deletion-carrying haplotypes (range 45.7%- 48.1%, Table 3).

**Table 3:** Transmission rate of deletion- and duplication-carrying haplotypes in HapMap YRI and EGCUT datasets combined.

CNV length	Deletions (no of del/all)	Duplications (no of dup/all)	All CNVs (no of del+dup/all)
**<10kb**	44.1%* (279/632)	**23.1%** (6/26)	43.3* (285/658)
**10–30kb**	**47.4%** (137/289)	**48.2%** (27/56)	**47.5%** (164/345)
**30–100kb**	**48.1%** (101/210)	**50%** (27/54)	**48.5%** (128/264)
**>100kb**	**45.7%** (43/94)	**51.7%** (30/58)	**48%** (73/152)
**All**	45.7%* (560/1225)	**46.4%** (90/194)	45.8%* (650/1419)

*Statistically significant (multiple-testing corrected p-value<0.05) deviations from the expected Mendelian transmission rate of 50%.

Transmission rate together with the number of transmitted variant-carrying haplotypes and the number of all transmission events (1 event/per locus/per child) for each of the non-overlapping CNV length intervals.

### Allelic variability within variant-carrying haplotypes in CNV loci

There were 56 regions in YRI families and 9 regions in EGCUT families in group B, where both parents had a CNV in the same locus (Table 2). These loci most likely represent common copy number polymorphic (CNP) regions in Yoruban and Estonian populations. In the combined dataset, in 34 (out of 39) unambiguously phased CNV loci both parents had a deletion-carrying haplotypes in the same region. In two unambiguously phased CNV regions there were both deletion- and duplication-carrying haplotypes present in different parents of the same family (e.g. 15q11.2 region in family Y045, S3 and S4 Figs) and in three regions both parents had duplication-carrying haplotypes (e.g. family Y004, S3 Fig), whereas in two of those regions there were different duplication-carrying haplotypes with alternative allelic copies present.

The ability of *PiCNV* to phase and differentiate between normal and CNV-carrying haplotypes with different allelic composition (Fig 1) allowed us to study the allelic variability within the discovered copy number gain-carrying haplotypes more extensively. As alternative allelic copies within CNV regions may modify severity of a phenotype including disease susceptibility, we aimed to determine the occurrence of alternative allelic copies *within* multi-copy haplotypes. We combined and studied all CNV regions in both HapMap YRI and EGCUT datasets in groups A and B where one or both parents of the same family had a duplication (cn = 2) or triplication (cn = 3)-carrying haplotypes in the same locus. In 207 out of 222 (93.2%) of such CNV regions in the combined dataset, informative polymorphic genotypes were present within the multi-copy haplotypes and it was possible to phase and differentiate *between* distinct normal and multi-copy haplotypes (Table 4). Furthermore, in 138 out of 222 (66.7%) of these CNV regions heterozygous genotypes were also present *within* the multi-copy haplotypes, allowing to define alternative allelic copies *within* these copy number gain-carrying haplotypes and demonstrating extensive allelic variability in multi-copy CNV regions in the human genome (Table 4 and S3 Fig).

**Table 4:** CNV regions in HapMap YRI and EGCUT parents where allelic variability between and within normal and copy number gain-carrying haplotypes can be deterministically differentiated.

	Dataset		
Haplotypes	HapMap YRI	EGCUT	Combined
Total number of copy number gain-carrying haplotypes in families	162	60	222
Informative markers present *between* normal and copy number gain-carrying haplotypes	**92%** (149)	**96.7%** (58)	**93.2%** (207)
Informative markers present *within* copy number gain-carrying haplotypes	**59.7%** (89)	**84.5%** (49)	**66.7%** (138)

In case informative polymorphic genotypes are present *between* haplotypes in an individual, copy number gain-carrying haplotypes (cn>1) can be deterministically distinguished from the normal single copy haplotypes (cn = 1). Furthermore, these informative genotypes can be used to establish the allelic composition and different allelic copies *within* copy number gain-carrying haplotypes.

### Putative *de novo* copy number variants in HapMap YRI and EGCUT families

In group C there were 27 CNVs (21 and 6 in HapMap YRI and EGCUT families, respectively) that were identified as putative *de novo* copy number alterations in offspring (Table 2). Out of these 27 putative *de novo* CNVs, 20 were deletions and 7 duplication events (S3 Table). As determined by *PiCNV* algorithm and confirmed by manual inspection, in four (out of five) unambiguously phased *de novo* CNV regions in HapMap YRI families new variants appeared on maternally and in one region on a paternally inherited chromosome. In EGCUT families, the emergence of the new variant was detected on the maternally inherited chromosome in one (out of four) unambiguously phased *de novo* CNV regions and on paternally inherited chromosomes in three regions (Fig 4).

## Discussion

We have developed a computational algorithm *PiCNV*, that uses genotype and copy number estimates from SNP genotyping data to determine the exact allelic composition and transmission of normal and copy number variable haplotypes in CNV regions in nuclear families. The algorithm was tested in two independent family-based datasets typed on different genome-wide SNP genotyping platforms.

Despite the decreasing sequencing costs and fast growing number of sequencing-based studies, SNP genotyping microarrays are still extensively used in genetic studies of disease susceptibility and in clinical diagnostics. In addition to SNP variants, absolute copy number estimates are often inferred from these genotyping datasets for genetic association studies. Determination of distinct parental haplotypes and allelic variability of the haplotypes at CNV regions in trios or larger nuclear families would specifically allow to study the transmission and contribution of individual haplotypes (and allelic copies) to phenotypic traits, including disease susceptibility.

To our knowledge, *PiCNV* is the first computational method that can deterministically phase null, mono-, di-, tri- and tetraploid genotypes and infer the allelic composition within normal and copy number gain-carrying haplotypes in CNV regions. *PiCNV* does not call CNVs itself but works with widely used algorithms such as PennCNV (*infer_snp_allele*.*pl*), QuantiSNP and Fawkes (from the Birdsuite package), that have specifically been developed for CNV calling [33, 34, 47]. In addition to copy number estimates, these programs can infer the total allelic composition at every genotyped marker within called CNVs which can be as accurate as in case of conventional two-letter genotypes [33]. Similar to conventional phasing algorithms that rely on two-letter SNP genotyping data [53–55], our algorithm uses the laws of Mendelian inheritance to automatically resolve the haplotype distribution in a family by looking at the allelic composition (SNP and CNV genotypes) of all family members and testing through all possible allelic combinations for each studied CNV region. Contrary to other family-aware phasing algorithms that avoid inheritance scenarios inconsistent with Mendelian inheritance [53, 56], *PiCNV* intentionally tries through the simplest non-Mendelian transmission scenarios (*de novo* deletions and duplications of parental haplotypes, uniparental iso- and heterodisomies), if the haplotype distribution cannot be explained by Mendelian inheritance scenarios. According to testing in HapMap YRI and EGCUT datasets, *PiCNV* can unambiguously determine the haplotypes and inheritance scenario for vast majority of CNV regions in families (93.6% of all CNV regions; Table 2). In case of complex CNV regions (e.g. multiple CNV-carrying haplotypes in parents) or if only monomorphic markers are present within a CNV region and the simplest haplotypes and Mendelian inheritance scenarios cannot be applied to explain the allelic composition in all family members, *PiCNV* is unable to determine a single set of correct haplotypes and inheritance scenarios and it will suggest several equally possible haplotypes and Mendelian and/or non-Mendelian transmissions (6.4% of all CNV regions). Accordingly, in both analysed datasets we observed that haplotype phasing was more efficient and resulted in one or very few equally possible parental haplotypes (and consequent Mendelian and non-Mendelian transmission events) in CNV regions where only one CNV-carrying haplotype was present in either parent (group A) compared to multiple CNV-carrying haplotypes in parents (group B, e.g. S2b Fig) and *de novo* CNV regions (group C, e.g. S2d Fig). And while the issue of multiple plausible Mendelian inheritance scenarios could be possibly solved by genotyping and computationally phasing of additional first degree relatives in the corresponding families, multiple equally possible non-Mendelian inheritance scenarios could be avoided by prioritising non-Mendelian events by their theoretical or estimated genomic frequencies.

In both datasets, we observed that CNV-carrying haplotypes were transmitted less frequently than normal haplotypes with the lowest rate (44.1%) detected for short (<10kb) deletion-carrying haplotypes (Table 3). Although the phenomenon might be expected in case of some high penetrance CNVs associated with severe disease phenotype, it has very briefly been investigated in healthy individuals [57, 58]. It has been suggested that such bias could be more pronounced for larger deletion variants interrupting genes of vital importance, consequently being more likely under stronger (prenatal) selection [1, 46, 49, 59–61]. Similar tendency was observed for longer (>10kb) deletions-carrying haplotypes in this study (Table 3). The opposite effect (even though statistically not significant) of slightly increased transmission rate of longer (>100kb) duplications observed could possibly be explained by contribution of duplications in providing means for functional redundancy [4, 62] and also in facilitating exon shuffling [63], gene fusion and gene duplication [64–66]. By generating new functional genes, duplication events may be important mechanism for long-term evolutionary changes in human and thus under positive selection [1, 3, 9, 67–69]. However, only very cautious interpretation of these results should be considered and larger studies with high-resolution techniques are required to confirm and further investigate these phenomena.

In addition to inherited variants *PiCNV* detected 27 putative *de novo* CNVs (Tables 2 and S2). We say ‘putative’ because even if unambiguously phased and validated by other experimental methods, such variants might be not true germline de novo mutations but instead somatically deriving cloned mutations or artefacts often observed in cell-line material [49, 70–72]. *De novo* CNVs might also ‘appear’ due to complex haplotype composition of a studied family in a given locus, e.g. in CNP loci [59, 73] where haplotypes with 0 and 2 copies are combined in one parent (e.g. 2q34 region in individual NA19092, S4 Fig) leading to incorrect calling of *de novo* variants from unphased CNV data and thus highlighting the relevance of phasing of the exact parental haplotypes. Additionally, similarly to the approach used by Kirov and colleagues [74], in the presence of haplotype-informative SNPs, *PiCNV* can determine on which parental chromosome the *de novo* aberration had occurred and in case of duplications, whether the event was inter- or intra-chromosomal (Fig 4b).

Deterministic phasing of haplotypes in families allowed us to elucidate the allelic variability within copy number variable loci. We found that in the majority of copy number gain regions it was possible to accurately determine and differentiate the alternative allelic copies *between* the single-copy and multi-copy haplotypes and also *within* the multi-copy haplotypes, suggesting a relatively wide variability in allelic composition within copy number gain CNVs (Table 4). Such true ‘CNV genotyping’, determination of the exact allelic composition within CNV regions, is necessary considering that many CNVs credibly associated with disease phenotypes are multi-allelic [5, 19, 75]. Alternative allelic copies present within CNV regions can modulate the severity of a given disease phenotype, partly explaining the low penetrance of most CNV loci (especially duplications) associated with disease so far.

In summary, we have developed a novel algorithm *PiCNV*, enabling to accurately determine the haplotype and allelic composition of CNV regions in family-based SNP genotyping datasets. The algorithm proved as a valuable tool to resolve the haplotype distribution in CNV regions and to follow the transmission of distinct haplotypes in offspring. Phased haploid copy number estimates (together with known parent-of-origin information) and the exact allelic composition of each haplotype allows to look ‘inside’ the CNVs and explicitly consider different allelic copies present in all studied individuals (e.g. cases and controls). Determination of allelic composition within CNVs provides new possibilities for studying CNVs in association with different phenotypic traits, including disease.

## Materials and Methods

### EGCUT cohort

All participants of this study were adult individuals from the Estonian population-based cohort [76] with no reported severe developmental disorders. All participants gave written informed consent and the study was approved by the Research Ethics Committee of University of Tartu. DNA extracted from peripheral blood was obtained from the Estonian Genome Center of University of Tartu (Tartu, Estonia; www.biobank.ee/en). These samples were genotyped by using Illumina (San Diego, CA, USA) Infinium HumanCNV370 chips at the Estonian Biocentre Genotyping Core Facility (Tartu, Estonia) according to the manufacturer's instructions. Raw microarray data was scanned with Illumina BeadStation and data normalisation was performed with the Illumina GenomeStudio software.

### Data for the YRI HapMap cohort

Illumina (San Diego, CA, USA) Infinium Human1M-Duo normalised microarray data generated by Itsara *et al*. [49] for 30 Yoruba Nigerian mother-father-child trios from the International HapMap Project [50, 51] were downloaded from NCBI Gene Expression Omnibus (http://www.ncbi.nlm.nih.gov/geo, GEO accession no GSE16896).

### CNV calling and filtering

Normalised microarray signal intensity data for both cohorts was analysed with PennCNV [47] (2009Aug27 v.) and QuantiSNP [33] (v.2) programs to call putative CNVs in each individual. Settings and parameters suggested by authors were used for both algorithms together with the ‘genomic wave’ adjustment for the signal intensity data. Additionally, with PennCNV we used separate B allele-frequency files (PFB-file) for both datasets—for the EGCUT dataset we used general Estonian population-based dataset [76] as the reference (n = 1000) and for HapMap YRI we used PennCNV’s default PFB-file based on HapMap samples. As a quality control measure, we checked that all samples met the following quality criteria calculated by the PennCNV program: LRR_SD≤0.25, BAF_SD≤0.05, BAF_DRIFT≤0.002 and GCWF≤|0.04|. Raw CNV calls from PennCNV and QuantiSNP were then merged (as intersection, for each individual separately) with custom PERL script and only CNVs that were similarly called (same type of overlapping copy number change—gain or loss) were considered. As CNV calls from one algorithm can possibly have many false positive calls, considering only CNVs called by more than one independent algorithm will minimise the number of false positive CNVs [77–79]. From the resulting list of CNVs we filtered out CNVs i) called on X/Y chromosomes; ii) shorter than 1000 bp in length; iii) with QuantiSNP log Bayes Factor (LBF) less than 5. Throughout this study we used the NCBI Build 36/hg18 assembly coordinates of the human reference sequence.

### CNV confirmation in the HapMap YRI dataset

In order to achieve high-quality CNV dataset, we confirmed our HapMap YRI CNV calls with an independent set of validated CNV calls for the same HapMap YRI individuals. CNV calls generated and confirmed with custom Affymetrix (Emeryville, CA, USA) high-resolution microarrays (with 32 million unique oligonucleotide probes for CNV discovery and 800,000 unique probes for CNV confirmation) by Matsuzaki *et al*. [52] were downloaded from http://genomebiology.com/2009/10/11/R125/additional. CNVs called by us and CNVs called and confirmed by Matsuzaki *et al*. [52] were compared for each individual separately (custom PERL script) and only CNVs that were called in both datasets were considered for further analyses.

### Visual confirmation in the EGCUT dataset

To ensure high-quality of the EGCUT CNV dataset, CNVs detected by PennCNV and QuantiSNP algorithms were further visually confirmed with Illumina Genome Viewer. For each CNV locus, signal intensity data for all corresponding family members was loaded simultaneously and visually inspected to confirm CNV calls and family members with no CNV call. CNV regions containing no visually detectable CNVs (or CNVs not called but visually distinguishable) were excluded from the further analyses.

### Converging CNV regions in families

All technically/visually confirmed CNVs were then converged into distinct CNV regions in families. We excluded those family-wise CNV regions in which any member of the corresponding family had any raw (unfiltered) CNV calls made by only one calling algorithm (custom PERL script), so that in each resulting CNV region at least one member of the respective family had a confirmed CNV while family members with normal diploid copy number (CN = 2) were proven to have no variant calls made by neither of the calling algorithms in the given region.

### Experimental validation of CNVs in the EGCUT dataset

In the EGCUT dataset (for which the DNAs were available to us), 20 CNV regions were selected for experimental validation by quantitative real-time PCR (qRT-PCR) in all members of the respective families (S1 Table). First, we randomly selected 12 loci where in the corresponding families at least one parent had a CNV call. Additionally, all eight putative *de novo* CNV regions were selected for validation. Considering all parents and siblings in the corresponding nuclear families, that summed up to 94 qRT-PCR assays. Out of those, 34 were regions with visually confirmed CNVs and 60 were regions with visually confirmed normal diploid copy number (CN = 2) in assumingly CNV-less members of the corresponding families. DNA samples of corresponding families were obtained from the Estonian Genome Center of University of Tartu. QRT-PCR primers were designed with the qRTDesigner tool (http://bioinfo.ut.ee/qrtdesigner) within each selected region. Universal controls with confirmed diploid copy number of two were used in each validation assay. QRT-PCR reactions were performed in a total volume of 10 μl consisting of 5× Hot FIREPol EvaGreen qPCR mix (Solis BioDyne), 10 ng of genomic DNA and 200–400 nM primers. Triplicate single-plex reactions were run on ABI Prism 7900HT real-time PCR system (Applied Biosystems) using the following amplification conditions: denaturation at 95°C for 15 min, followed by 40 cycles of denaturation at 95°C for 15 s, annealing at 60°C for 20 s and elongation at 72°C for 20 s. Absolute quantitation results were normalised to internal standard gene ALB and a reference DNA pool compiled of unrelated Estonian samples (n = 50). Out of 20 loci selected, four loci failed due to unsuccessful primer design. In total (also considering *de novo* CNV loci), out of 76 validated regions, 20 individual CNV calls were proven to be true positives. Out of these, 15 were deletions and 5 were duplications. 48 loci were proven to be true negatives, i.e. there were no CNVs in those regions in the corresponding family members and those regions were confirmed to have expected normal diploid copy number (CN = 2). Six regions were proven to be false positives and two as false negatives and these CNV loci were excluded from the subsequent analysis.

### Computational phasing of CNV regions in families

As the first step, regular two-letter and CNV genotypes were collected from the QuantiSNP output for each family member at each CNV region. If there were several individuals in the family with a CNV in the analysed region, the intersection region of these individual CNV calls was used (requiring at least 3 ‘shared’ markers) and markers that were not part of the consecutive CNV region observed in each family member with a CNV call were discarded. Next, markers that had low-confidence genotype calls (regular genotype/CNV genotype call probability <0.95) or where the call could not have been made (‘NC’ genotypes) and markers that did not have informative genotypes for haplotype phasing in the studied region were filtered out. Next, *PiCNV* generated the comprehensible list of all possible elementary inheritance events for all mother-father-child trios for all markers in a given CNV region. An elementary inheritance event describes unambiguously both the allelic composition of a given marker in parent genotype and how specific haplotypes from both parents combine and produce the genotype of a zygote, additionally to possible Mendelian transmissions also allowing the simplest non-Mendelian events (*de novo* deletions and duplications and uniparental iso- and heterodisomies). Next, a three-step algorithm was used. Firstly, the best combination of haplotypes in both parents was searched by minimising the number of unexplainable genotypes and the number of non-Mendelian events in children. Secondly, the best gamete formation schemes in each family were searched, also by minimising the number of unexplainable genotypes and the number of non-Mendelian events in children. Gamete formation scheme describes unambiguously how allele(s) in one parent form the genotype of a gamete (haplotype) by unmodified transmission of parental allele(s). As in case of Mendelian transmissions, considered non-Mendelian events in a gamete formation had to be consistent for all genotypes within a CNV for each mother-father-child trio, but could vary between different parents-child trios if more than one child was available. Finally, the best phased genotype(s) for each marker that were consistent with allelic combination and gamete formation were searched, also minimising the number of unexplainable genotypes and the number of non-Mendelian events in a given family and presented as the result. If there were no haplotype-informative markers within a CNV region and the simplest haplotypes and Mendelian inheritance scenarios did not explain the allelic composition in all family members, it was not possible to determine a single set of correct haplotypes and inheritance scenarios and several equally possible haplotypes and Mendelian and/or non-Mendelian transmissions were suggested as the result.

On an Intel Xeon 2.27GHz computer (running 64-bit CentOS Linux) the whole procedure took about 2 minutes (using 2.5GB of RAM) for 34 EGCUT families genotyped on low-resolution Illumina HumanCNV370K array and less than 9 minutes (max 6.4GB of RAM) for 30 HapMap YRI trios genotyped on high-resolution Illumina Human1M array.

### Transmission of normal and CNV-carrying haplotypes

This analysis was carried out only in unambiguously phased CNV regions in group A. We decided not to include the small number of unambiguously phased CNV regions in group B for several reasons: i) to keep the transmission analysis as straightforward as possible; ii) if both haplotypes in one (or both) parents are variant-carrying, inheritance of normal haplotypes is not possible at all; iii) overall phasing efficiency in group B was much lower compared to group A. In each unambiguously phased CNV region in group A we counted the number of transmissions of normal and CNV-carrying haplotypes from the parents carrying a CNV to their offspring and these counts were summed over all CNV regions in the combined dataset. This was repeated for deletion and duplication CNV loci separately, also considering their length. Deviance from the expected 50:50 transmission (no preference in inheritance of normal or CNV-carrying haplotypes) was tested with the Pearson’s chi-square test in the statistical package R (ver. 2.13.0; http://www.R-project.org).

### Allelic variability within copy number variant-carrying haplotypes

Unambiguously phased parental haplotypes in CNV regions where one (group A) or both (group B) parents of the same family had a duplication (cn = 2) or triplication (cn = 3)-carrying haplotypes were studied. For each respective CNV region and parent, the number of polymorphic (normal and CNV) genotypes present between the normal and copy number gain-carrying haplotypes (e.g. SNP2 and SNP3; Fig 1) and within the copy number gain-carrying haplotypes (e.g. SNP7; Fig 1) was calculated. If there were one or more polymorphic genotypes present within the copy number gain-carrying haplotypes, the corresponding alleles were considered as different and if there were no polymorphic genotypes present, the corresponding alleles were considered to be identical.

## Supporting Information

S1 Fig Step-by-step description of data analysis and filtering steps. Based on the occurrences of CNVs in parents in every family, all CNV regions and corresponding transmission events were divided into three groups: group A—CNV regions where in any given region only one parent had a CNV (marked as ‘X’ on illustrative homologous chromosomes) that might or might not have been transmitted (marked as ‘?’) to offspring; group B—CNV regions and transmission events where both parents had a CNV in the same locus that might or might not have been transmitted to their offspring; group C—putative *de novo* CNVs where parents did not have any CNVs, but at least one child had a CNV in the corresponding locus.
(TIF)

S2 Fig Examples of copy number gain and *de novo* CNV regions for which unambiguous phasing of exact parental haplotypes and/or following their transmission in offspring was not automatically possible, resulting is several equally possible transmission scenarios. (A) A 69 kb-long duplication region on chromosome 2 in family T06 that (due to two equally possible paternal haplotype combinations and corresponding transmission scenarios) was not unambiguously phased. Paternal genotypes that can be distributed differently on two distinct combinations of paternal haplotypes are indicated with dashed rectangles. (B) A 10 kb-long multi-copy CNV region on chromosome 10. In case there are only monomorphic uninformative genotypes present in parents (e.g. family Y005), several equally possible haplotype configurations and Mendelian transmission scenarios are possible. (C) In case there are haplotype-informative genotypes present in parents (as for the same CNV region in families Y105 and Y042), it is possible to determine the exact parental haplotypes and follow their transmission in the offspring. (D) A family with a 103 kb-long *de novo* deletion on chromosome 1 found in an offspring. Due to undistinguishable haplotypes in parents it is not possible to determine, on which parental chromosome the *de novo* deletion has occurred.
(TIF)

S3 Fig A 155 kb-long copy number polymorphic (CNP) region on chromosome 15:19811954..19967627 with normal and both deletion- and duplication-carrying haplotypes. Considering the allelic composition *within* these duplication-carrying haplotypes, several different allelic copies seem to be present within this region. Heterozygous genotypes *within* the duplication-carrying haplotypes that enable distinguishing the allelic copies within these haplotypes are indicated with dotted rectangle.
(TIF)

S4 Fig An interesting CNV region at chromosome 2:213922542..213939290. In family Y040, the mother has a 17 kb-long copy number gain (diploid copy number CN = 4) and the father has normal diploid copy number (CN = 2) in the same locus. Interestingly, their child had a copy number gain with diploid copy number of four (CN = 4). Given the allelic composition within that CNV region in this family, the only appropriate Mendelian inheritance scenario was proposed whereby the child had inherited a duplication-carrying haplotype from her mother and also from her father. This is demonstrating a highly polymorphic CNV locus with 0, 1 and 2 copies (including several different allelic copies) present on homologous chromosomes and haplotypes with 2 and 0 copies combined in the corresponding father.
(TIF)

S1 Table Quantitative real-time PCR validation results. (XLSX)

S2 Table Putative *de novo* CNVs in HapMap YRI and EGCUT families. (XLSX)

S3 Table List of analysed CNV regions in HapMap YRI and EGCUT families together with the phasing and haplotype information (*PiCNV* output) for each family member. (XLSX)
